# Covalent Chemical Tools for Profiling Post-Translational Modifications

**DOI:** 10.3389/fchem.2022.868773

**Published:** 2022-07-04

**Authors:** Benjamin Emenike, Ogonna Nwajiobi, Monika Raj

**Affiliations:** Department of Chemistry, Emory University, Atlanta, GA, United States

**Keywords:** chemoselective, posttranslational modifications, proteins, chemical probes, covalent bond

## Abstract

Nature increases the functional diversity of the proteome through posttranslational modifications (PTMs); a process that involves the proteolytic processing or catalytic attachment of diverse functional groups onto proteins. These modifications modulate a host of biological activities and responses. Consequently, anomalous PTMs often correlate to a host of diseases, hence there is a need to detect these transformations, both qualitatively and quantitatively. One technique that has gained traction is the use of robust chemical strategies to label different PTMs. By utilizing the intrinsic chemical reactivity of the different chemical groups on the target amino acid residues, this strategy can facilitate the delineation of the overarching and inclusionary roles of these different modifications. Herein, we will discuss the current state of the art in post-translational modification analysis, with a direct focus on covalent chemical methods used for detecting them.

## 1 Introduction

**FIGURE 1:**
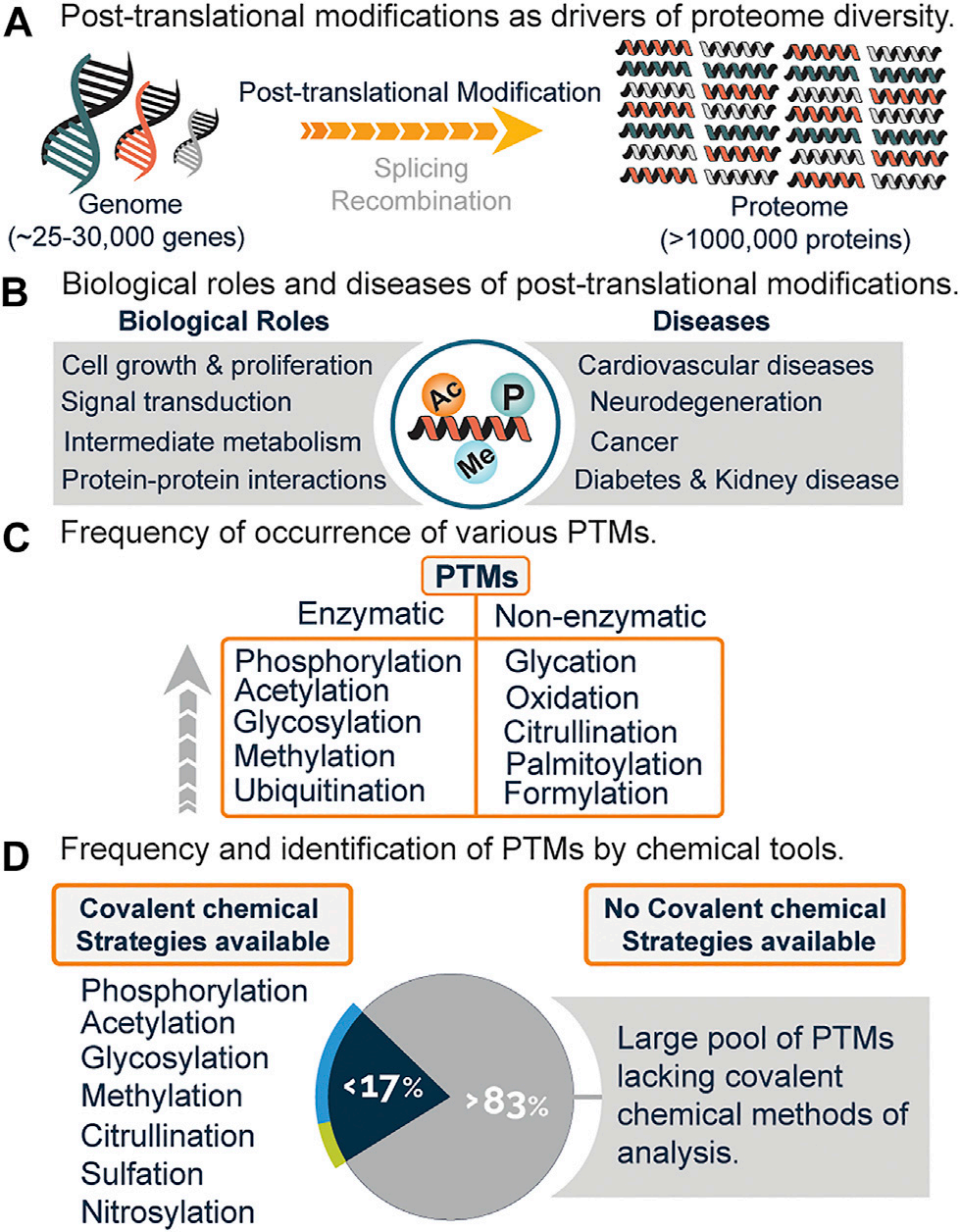
**(A)** Increased protein diversity due to post-translational modifications (PTMs), alternative splicing, and recombination. **(B)** Biological roles and diseases associated with PTMs. **(C)** Frequency of various PTMs and mode of generation i.e., enzymatic or non-enzymatic. **(D)** Estimated PTMs that have covalent chemical profiling strategies and the lack of chemical strategies for a significant percentage of PTMs.

The completion of the human genome project gave rise to a plethora of biological information which needed translation, with one being the discrepancy between genes and gene products. Of the 25,000–30,000 human genes identified, only about 2% code for proteins, with the total number of proteins estimated to be over a million (Figure 1A) (Lander et al., 2001). A major biological event that accounts for such discrepancy, in addition to alternative splicing and genomic recombination, is post-translational modification (PTM).

Post-translational modifications encompass the enzymatic or non-enzymatic addition of diverse functional groups onto the side chain or main chain of amino acid residues. These modifications regulate vital cellular processes such as signal transduction, cell growth and proliferation, metabolism, protein-protein interactions, and a host of biological functions (Figure 1B) (Deribe et al., 2010; Li X. et al., 2013; Van Der Laan and Maiorano, 2014). Anomalous expression is linked to diseases such as diabetes, cancer, neurodegeneration, cardiovascular diseases, and kidney diseases (Figure 1B) (Tomonaga et al., 2004; Ren et al., 2014; Smith and White, 2014; Chatterjee and Thakur, 2018).

Presently, over 620 types of posttranslational modifications have been identified experimentally, with phosphorylation, acetylation, glycosylation, and methylation leading the chart as the most abundant (Figure 1C) (Khoury et al., 2011). While there is an increasing demand to discover novel PTMs, a lot of effort has been channeled towards the analysis and study of already known PTMs (Pinkse et al., 2011; Bremang et al., 2013; Kaitlyn et al., 2013; Mertins et al., 2013).

**FIGURE 2:**
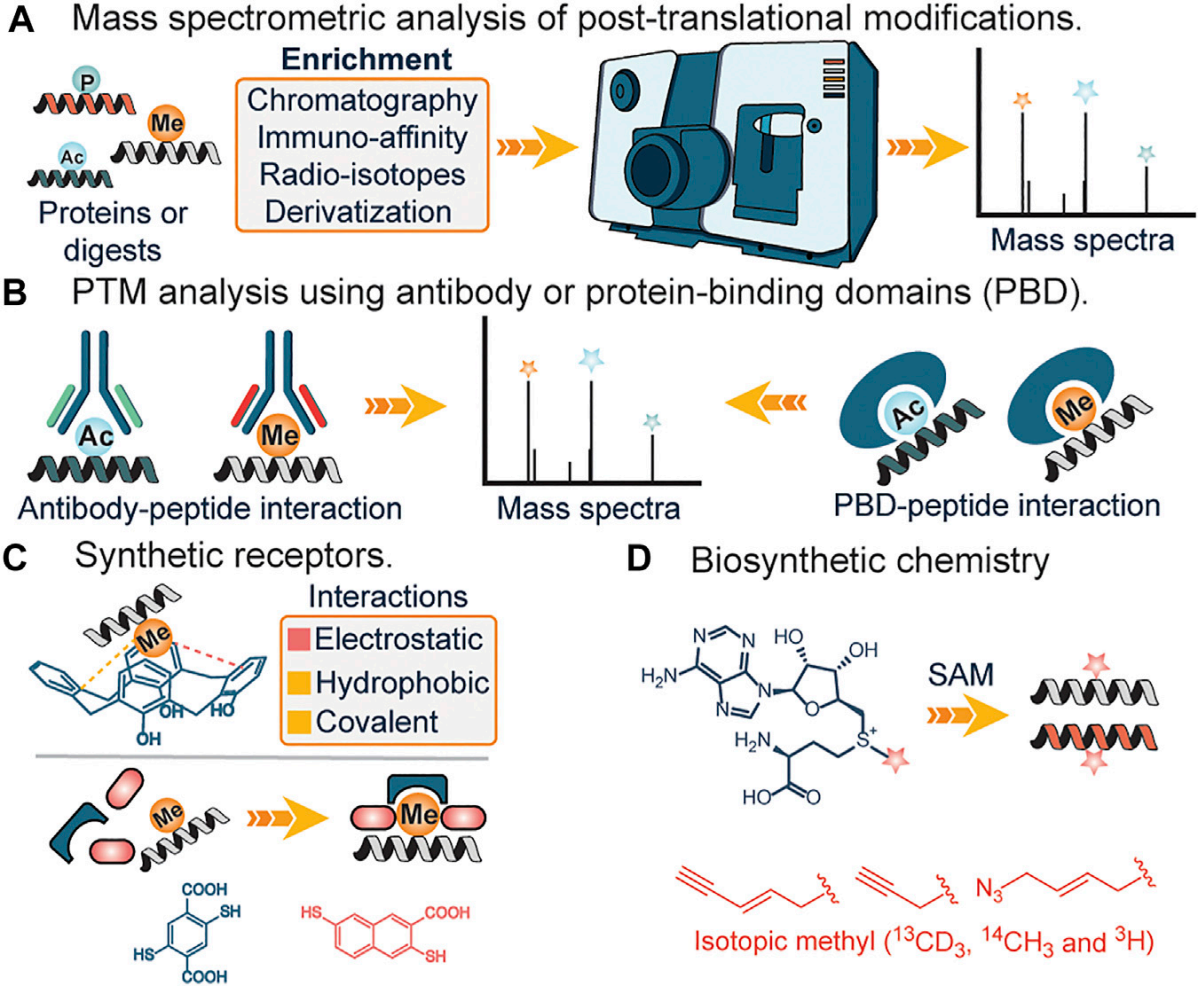
**(A)** Analysis of PTMs using mass spectrometry. **(B)** Analysis of PTMs using antibody and protein-binding domains. **(C)** Interactions of synthetic receptors and PTMs (methylation). **(D)** Biosynthetic analogs of s-adenosylmethionine for affinity tag or isotopic tag labeling of lysine methylation.

Recently, chemical proteomics is emerging as a new Frontier towards the global profiling of PTMs in the proteome. This strategy generally relies on the chemical transformation or covalent modification of various PTMs with highly selective chemical probes. With this field still in its infancy, it is believed that the use of chemical tools will not only serve to complement existing methods (Figure 2) but might be the gold standard for studies related to PTM analysis and cross-talk. Out of the very many posttranslational modifications that are known, only a few have been identified using covalent chemical strategies, while a whopping percentage is yet to be chemically modified (Figure 1D). Herein, we will briefly discuss the current methods for analyzing PTMs, followed by a detailed overview of the current covalent chemical repertoire for analyzing the PTMs. Lastly, we will discuss the prospects of this field.

## 2 Techniques for Identification of Posttranslational Modification’s

**Mass spectrometry:** Mass spectrometry (MS) has transformed the analysis of biomolecules and it is practically preposterous to imagine the analysis and identification of proteins and their modifications without this tool. In the field of proteomics, MS-based technique is traditionally stratified into top-down or bottom-up proteomics, with the top-down approach more efficient in identifying and characterizing the type and site of PTMs, while the bottom-up approach involves degradation of proteins into smaller fragments before MS analysis, aids in derivatization and identification of buried PTM sites in a complex protein structure. Due to the low abundance of PTMs, prior enrichment of samples from a complex mixture using either chemical derivatization (Mertins et al., 2013), affinity reagents (Guo et al., 2014; Cao and Garcia, 2016) or radioisotope-based approach (Bremang et al., 2013) is often required. This serves to reduce sample complexity. Furthermore, issues such as false positives due to different parents masses having similar mass-charge values are often observed (Zhang et al., 2004).

**Antibodies and Protein Binding Domains.** The use of highly specific antibodies against PTMs represents another strategy for elucidation of PTMs. This generally involves the engineering of different antibody domains specific to target antigens such as acetyl or methyl groups (Figure 2B). Subsequently, immunoaffinity pull-down of lysates is applied to extract the target post-translationally modified peptides (Ivanov et al., 2004; Guo et al., 2014). Similarly, protein-binding domains (PBDs) represent a slightly different approach and often serve as a suitable alternative when no pan-specific antibody for a target PTM is available (Figure 2B). Naturally occurring or engineered protein-binding domains against methylation represent a popular application of this strategy. In 2013, Gozani *et al.* utilized an engineered three malignant brain tumor repeat (3 MBT) of L3MBTL1 for detecting and enriching methylated lysines (Kaitlyn et al., 2013). These affinity-based strategies are unable to distinguish different methylation states of lysine, suffer from lack of specificity and irregular binding to targets due to the presence of adjacent PTMs at the site of interest. Furthermore, the issue of batch-to-batch irreproducibility is also of utmost concern (Bock et al., 2011; Nishikori et al., 2012; Busby et al., 2016).

**Synthetic receptors.** Unlike their biological counterparts, synthetic receptors have found great application in analyte detection. By utilizing synthetic or dynamic combinatorial chemistry approaches, scaffolds that bind to target PTMs are used as artificial receptors. In 2010, Hof and co-workers demonstrated the feasibility of using deprotonated anionic tetrazolates and macrocycles such as calixarenes, as potential receptors for hydrophobic quaternary ammonium cations such as the side chain of trimethyllysines in peptides and proteins (Daze and Hof, 2013) (Figure 2C). The design mimicked aromatic cages found in proteins, with the major drivers of recognition being electrostatic and non-classical hydrophobic interactions. Since then, diverse versions of synthetic receptors have been introduced (Corbett et al., 2008; Li C. et al., 2013; Peacock et al., 2016). Corbett *et al.* developed a dynamic combinatorial library of macrocyclic receptors targeting a variety of quaternary ammonium compounds. Similarly, Li *et al.* and Peacock *et al.* utilized a negatively charged carboxyatopillar [5] arene and tetracyanoresorcin [4] arene receptor to bind to basic and positively charged amino acid residues and trimethyllysine peptides. Just recently, Waters *et al.* developed the “imprint and report” technology that utilizes building blocks that form reversible covalent bonds as a templating platform to facilitate a host-guest interplay which subsequently leads to interpretable signals for different methylated states of lysine and arginine (Harrison et al., 2021) (Figure 2C).

The robustness of synthetic receptors makes them attractive candidates as stationary phases for chromatography. Boronate affinity chromatography which utilizes the reversible covalent association of diol-containing carbohydrates with boronic acids is used extensively for clinical quantification of glycated hemoglobin and separation of glycoproteins from complex mixtures (Hage et al., 2012). Synthetic receptors such as weak anion exchange resin (WAX) (Robinson et al., 2014) have been used for the enrichment of sulfated tyrosine. Before weak anion exchange, a carbamylation reaction was employed to neutralize positive charges on primary amines without the loss of the labile sulfate group. The carbamylated sulfopeptides bind more efficiently to WAX resin relative to nonsulfated peptides and this approach has been used successfully to identify a sulfated peptide, DDGDGsYIEIIPR, corresponding to sulfation at Tyr 1,513 of enzymatically digested factor V.

The immobilized metal ion affinity chromatography uses the reversible coordination of metal cations like Zn_2_^+^, Cu_2_^+^, Ni_2_^+^, or Fe_3_^+^ by biomolecular targets like amino acids, peptides, proteins, or nucleic acids (Hage et al., 2012). They are used extensively to purify proteins containing multiple histidine residues (His-tagged proteins). Balderrama *et al.* employed metal affinity enrichment to recognize Tyrosine sulfation (sTyr) which relies on the interaction between positively charged chelated metal ion and the negatively charged sulfate moiety of sTyr (Balderrama et al., 2011). Metal-oxide affinity chromatography typically employs beads of titanium dioxide as a stationary phase to retain acidic biomolecules, especially phosphorylated peptides (Beltran and Cutillas, 2012). Presently, artificial receptors are plagued with minimal selectivity over structurally similar analytes while some display a reduced affinity towards target ligands.

**Biosynthetic Chemistry.** Occasionally, biological pathways can be utilized to study posttranslational modifications. The central tenet involves the incorporation of chemically modified or radiolabeled cofactors that are active for rationally designed enzymes, in place of canonical ones (Rathert et al., 2008; Islam et al., 2012) (Figure 2D). A typical example is the incubation of cells with clickable or radiolabeled S-adenosylmethionine, a ubiquitous methyl donor in SAM-dependent methylation events. Labeled residues are subsequently characterized using mass spectrometry analysis. Similarly, this method was applied in lipidation profiling (Hang et al., 2007; Heal et al., 2008). In the first step, labeled myristic acid or palmitic acid analogs are delivered to cells in culture where they are converted to their active acyl-CoA form by endogenous acyl-CoA synthase activity; metabolic incorporation allows for subsequent enrichment and identification. Biosynthetic pathways have also been reported to identify the functions of varying other PTM’s including lysine succinylation. These methods utilize the Amber codon-suppression based mutagenesis approach for labeling proteins with an unnatural amino acid such as azidonorleucine by biological machinery followed by traceless Staudinger ligation that enables the formation of an amide bond between an azide and phosphinothioester (or phosphinoester) for the site-specific installation of different lysine acylation on proteins including succinylation (Wang et al., 2017). These studies afforded access to proteins with site-specific acylation (succinylation) to understand the functional roles of lysine succinylation. The drawbacks of biosynthetic chemistry approaches include poor membrane uptake of analogs, cross-reactivity with other amino acids, in addition to potential environmental contamination due to the use of radioactive analogs.

Although several methods have been reported for the identification of varying PTMs and those methods are very well-reviewed elsewhere (Nørregaard Jensen, 2004; Kirkpatrick et al., 2005; Silva et al., 2013; Shumyantseva et al., 2014; Leutert et al., 2021). In this paper, we focus mainly on the chemical methods that lead to the formation of covalent bonds with the PTMs for their characterization and identification.

## 3 Covalent Chemical Methods for Identification of Posttranslational Modification’s

### 3.1 Phosphorylation

Widespread interest in phosphorylation PTM has led to the development of numerous covalent chemical strategies for its analysis. A common theme among these methods involve, the selective enrichment of phosphopeptides from a complex mixture of peptides through a sequence of chemical reactions, followed by the subsequent analysis of isolated phosphopeptides by mass spectrometry.

**FIGURE 3:**
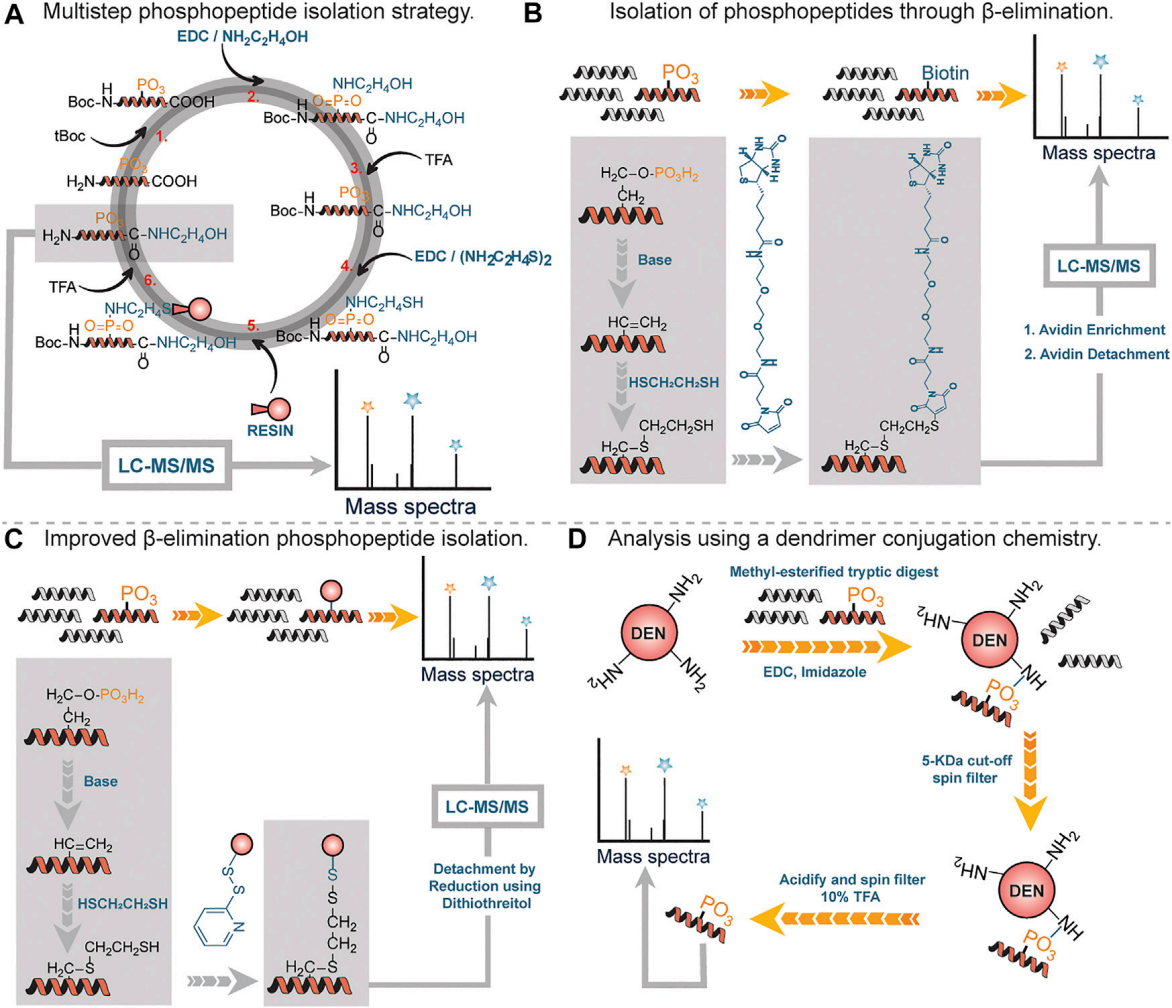
**(A)** Multistep approach to phosphopeptide isolation. **(B)** Isolation and analysis of phosphopeptides through β-elimination of terminal phosphate, followed by alkylation with biotin. **(C)** Improved β-elimination strategy with a release by disulfide bonds. **(D)** A 3-step reaction sequence for isolating phosphopeptide by using functionalized soluble dendrimers.

One of the first covalent chemical approaches was developed by Aebersold *et al.,* in 2001. They developed a multistep chemical method consisting of six reaction steps (Figure 3A) and applied it on tryptic digests containing various phosphorylated peptides (Zhou et al., 2001). First, the amino groups of tryptic digested peptides were protected using tert-butyl-dicarbonate (tBoc) chemistry (step 1). This was done to prevent potential intermolecular and intramolecular reactions in subsequent steps. Once the amino groups were protected, the carboxylic groups were protected using a carbodiimide-catalyzed condensation reaction with excess amines to form amides, and phosphoramidates, resulting from the modification of phosphates by the coupling reaction (step 2). Free phosphate groups were regenerated by acid hydrolysis (step 3, carboxylic acids remain protected), followed by a subsequent carbodiimide-catalyzed condensation reaction of regenerated phosphate groups with cysteamine (step 4). Internal disulfides of cysteamine were reduced prior to solid-phase capture of peptides through the reaction between the free sulfhydryl group of cysteamine with the iodoacetyl group on the resin (step 5). Acid cleavage of the phosphoramidate bonds led to the isolation of free phosphopeptides which were subsequently analyzed using LC-MS/MS. It is noteworthy to state that the carboxylate groups remained blocked at the end of the reactions (step 2). By utilizing various phosphopeptides and yeast cell lysates, Aebersold and coworkers demonstrated that their method was equally applicable in isolating serine-, threonine- and tyrosine-phosphorylated peptides from a highly complex peptide mixture.

By far, the most prevalent method for phosphopeptide analysis involves *β*-elimination of phosphate moiety under basic conditions by appropriate affinity tags (Figures 3B,C). The central principle is based on the fact that phosphate moieties on serine or threonine containing peptide residues are labile at high pH values (Fadden and Haystead, 1995; Jaffe et al., 1998). Under basic conditions, the phosphate moiety on phosphoseryl or phosphothreonyl residues can undergo *β*-elimination to form dehydroalanine, an alpha-beta unsaturated Michael acceptor which can readily react with a nucleophile such as ethanedithiol. Thiol containing modified residues are coupled to biotin through a maleimide or iodoalkyl linked biotin moiety (Figure 3B). Biotinylated peptides are enriched using avidin column chromatography followed by the release of enriched peptide under acidic conditions for analysis by MS (Oda et al., 2001). One major drawback associated with the use of biotin as an affinity handle is the inefficient recovery of tagged residues after affinity column chromatography. To address this difficulty, Chait *et al.*, utilized a thiol containing affinity reagent instead of biotin (McLachlin and Chait, 2003). Reduction with dithiothreitol led to the recovery of modified phosphorylated peptides (Figure 3C). While this approach was effective in addressing issues associated with recovery, it did lead to side reactions where unmodified cysteine residues were captured by the affinity tag (∼1–2%).

In 2005, Aebersold *et al.* described an improved strategy for phosphopeptide enrichment and subsequent analysis. This improvement arises simply from the capture of phosphopeptides using a one-step chemical reaction between soluble amine-functionalized polymer (Generation-5 polyamidoamine dendrimer) and phosphate moiety on peptides to generate phosphoramidates (Figure 3D). This reaction led to peptide conjugates that were physically larger than unmodified peptides, thus were readily separated from other peptides and excess reagents using a 5-KDa filtration spin column. Notably, this approach was applicable to all classes of phosphopeptides including phosphotyrosine containing peptides. Using this approach, Aebersold and coworkers identified over 150 phosphorylation sites (Tao et al., 2005).

### 3.2 Acetylation

Acetylation on both the lysine side chains and the N-terminus is a common protein modification that affects an estimated 80% of all human protein species to varying degrees. However, the enrichment of acetylated peptide residues is difficult because acetylated amines cannot be easily derivatized. Normally, the lysine acetylome is characterized and quantified by enrichment with antibodies against acetylated lysine residues and subsequent mass spectrometric analysis.

**FIGURE 4:**
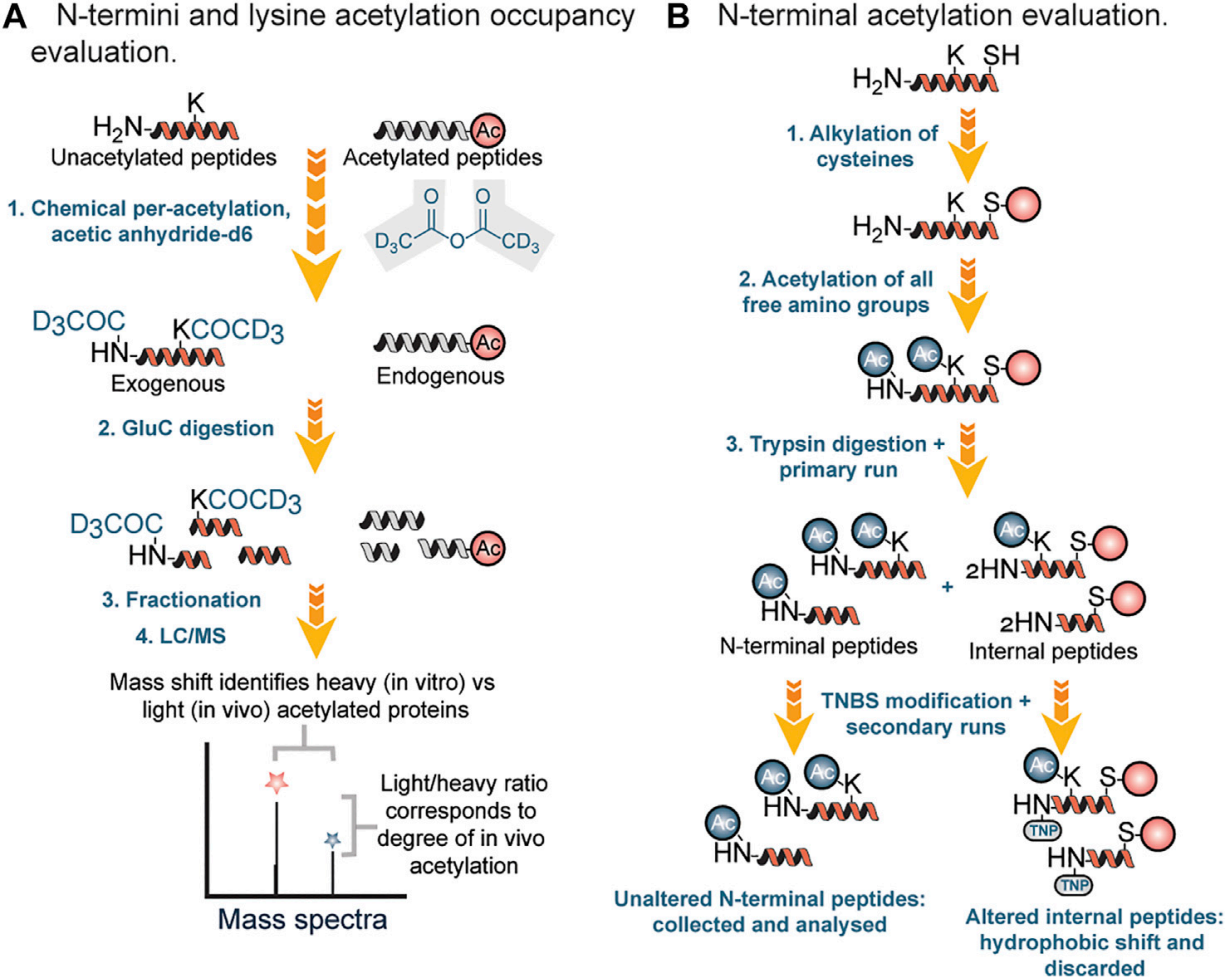
**(A)** Stoichiometry workflow for evaluation of N-terminal and lysine acetylation based on occupancy ratio. **(B)** Chemical and chromatographic steps for identification of N-terminal acetyl modified proteins.

Chemical methods of profiling acetylation often rely on the stoichiometric lysine or N-termini acetylation occupancy (i.e., the proportion occupied [acetylated] versus unoccupied [unacetylated]). Recently, several groups have reported methods for determining the occupancy of lysine acetylation sites, which measure the ratio of endogenously acetylated lysine to unmodified lysine (i.e., stoichiometry or occupancy) (Meyer et al., 2016). In stoichiometric experiments, mass spectrometric recordings determine the ratio of endogenous light acetyl groups to isotope-labeled heavy acetyl groups, which are generated *in vitro* by quantitative acetylation of all unmodified lysines after the cells have been harvested (Figure 4A). In this workflow, protein lysates were first incubated three times with acetic anhydride-d_6_ in order to acetylate unmodified lysine residues. Second, samples were digested with endoproteinase Glu-C, followed by fractionation of the proteolytic peptides by basic reverse phase chromatography. Finally, peptides were analyzed by LC-MS.

In 2003 Gevaert et al. described a diagonal procedure for the isolation of N-terminal acetylated peptides (Figure 4B). In this new process, free cysteine groups were first blocked by alkylation followed by acetylation of amino groups in proteins and then digestion with trypsin (Gevaert et al., 2003). After reverse phase fractionation (RP) of the generated peptide mixture, internal peptides were blocked using 2,4,6-trinitrobenzene sulfonic acid (TNBS). During the modification, internal peptides showed a strong hydrophobic shift and therefore segregated from the unchanged N-terminal peptides during a second identical separation step. N-terminal peptides were then collected specifically for further liquid chromatography (LC)-MS/MS analysis. Using this approach, Gavaert and coworkers were able to identify 264 proteins and 78 *in vivo* acetylated proteins in a cytosolic and membrane skeleton fraction of human thrombocytes.

### 3.3 Glycosylation

Of the various types of post-translational modifications, glycosylation is the most chemically and biosynthetically complex. The complexity of glycosylation results from two factors: the diversity of the building blocks and the diverse ways in which oligosaccharides can be built up within the cell. Furthermore, the non-templated biosynthesis generates enormous diversity and heterogeneity within the cellular glycome.

**FIGURE 5:**
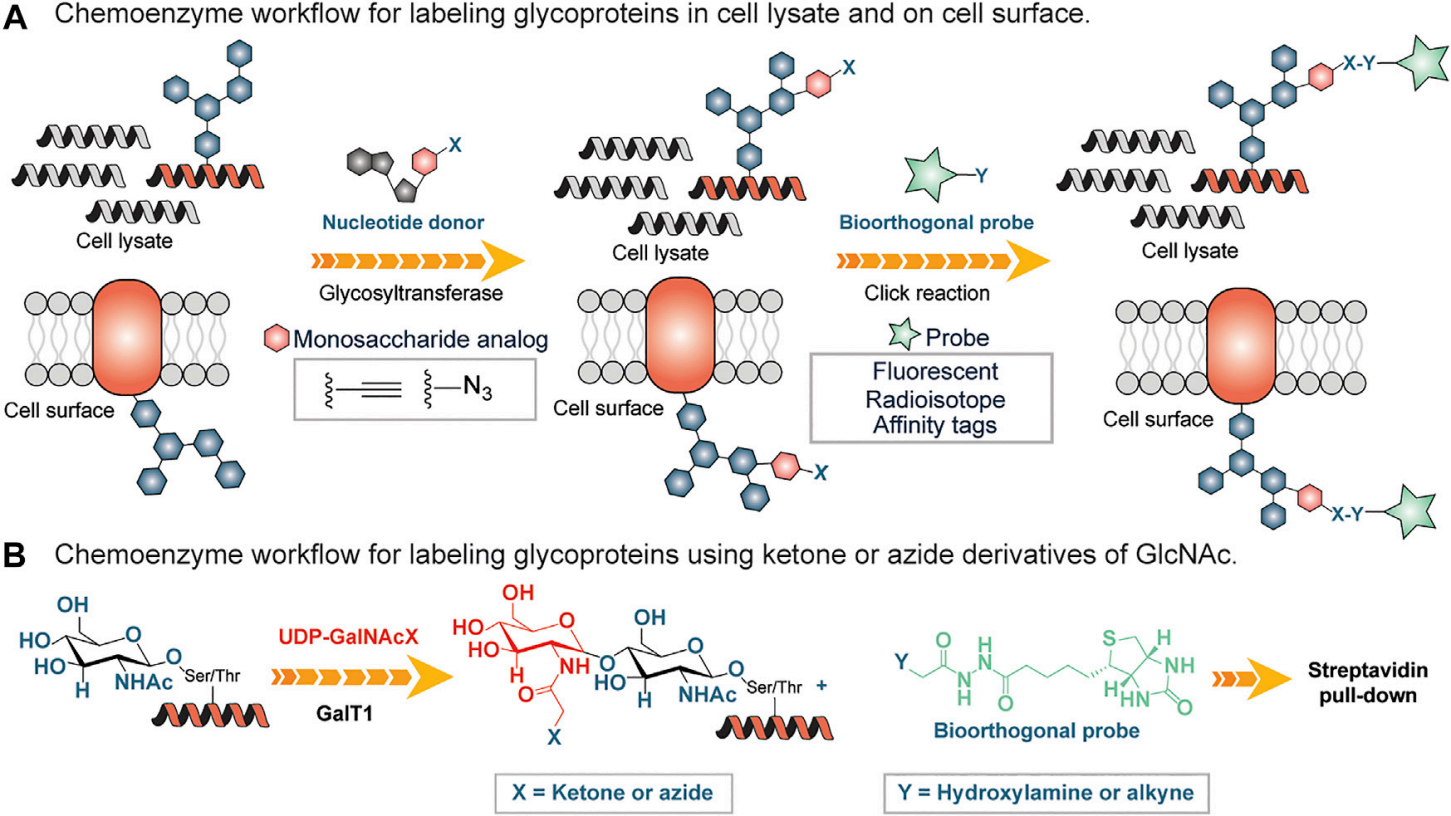
**(A)** Chemoenzymatic glycan labeling on cell lysates using a monosaccharide equipped with a reactive group (X) and transferred from a nucleotide sugar donor to a glycoprotein in the cell lysates or cell surfaces by a Glycosyltransferase. Y is derivatized with an affinity tag or fluorescent probe and subsequently enriches labeled glycoproteins. **(B)** Chemo-enzymatic labeling workflow using a mutated galactosyltranferase GalT1 to insert GlcNAc bearing ketone or azide handles, followed by enrichment using hydroxylamine or alkyne derivatized biotin tag.

Chemical methods have long been used as alternative tools for the detection of glycans. Many of these approaches are destructive (Rutland, 2008) involving the use of periodic acid and alcian blue to detect sialic acid and polysialic acid, respectively. Both the reagents oxidize sialic acid to decarbonylated sialic acid and cause permanent damage to the treated carbohydrate chains thus preventing downstream applications. Lately, there have been powerful approaches to glycan detection that are variations of originally developed methods (Kayser et al., 1992; Dube and Bertozzi, 2003). One widely used approach is based on chemoenzymatic glycan labeling (CeGL). In this approach, Bertozzi and coworkers used a recombinant glycosyltransferase to transfer a monosaccharide analog from a nucleotide sugar donor to a specific glycan acceptor directly in cell lysates or on the cell surface (Dube and Bertozzi, 2003). The transferred monosaccharide was equipped with a reactive handle that was further derivatized to incorporate a detection probe (Figure 5A).

Inspired by the work of Torres and Hart, who incorporated GalT1 radiolabeled Uridine diphosphate N-acetylglucosamine (UDP-[3H]-Gal) into N-acetylglucosamine (GlcNac) residues (Torres and Hart, 1984), Hsieh-Wilson and coworkers used a mutated form of *β*1,4-galactosyltransferase (GalT1 Y289L) to facilitate the transfer of the N-Acetylglucosamine (GlcNAc) with an unnatural chemical ketone handle (Khidekel et al., 2003) (Figure 5B). The ketone functional group was labeled with an aminooxy-biotin probe for enrichment by streptavidin conjugates followed by analysis using LC-MS/MS (Figure 5B). Subsequently, the substrate scope of this GalT1 mutant was expanded to include an azide-bearing UDP-GlcNAcs as a nucleotide sugar donor that led to the attachment of affinity tag by copper(I)-catalyzed azide-alkyne cycloaddition (CuAAC), thus reducing the unspecific labeling in crude cell lysates containing other aldehydes and ketones, a disadvantage in connection with the reactive ketone/aminooxy pair.

Recently, Hsieh-Wilson and colleagues developed a method to quantify O-GlcNAc glycosylation stoichiometry and to understand dynamics. In this procedure, GalT1 was used to incorporate a ketogalactose residue into O-GlcNAcylated proteins, which was then reacted with aminooxy-functionalized polyethylene glycol (PEG) mass tag (Rexach et al., 2010). In this way, tags with defined molecular weights were attached to O-GlcNAcylated proteins, and the stoichiometry of O-GlcNAc modifications was resolved by SDS-PAGE by simply examining mass-shifted bands.

Although CeGL methods are relatively new and have a wide range of applications, one of the greatest challenges for the future expansion of CeGL is the identification of additional glycosyltransferases that have strict acceptor specificity but also show promiscuity for modified nucleotide sugar donors that can be used for glycan labeling in a cellular system.

### 3.4 Methylation

**FIGURE 6:**
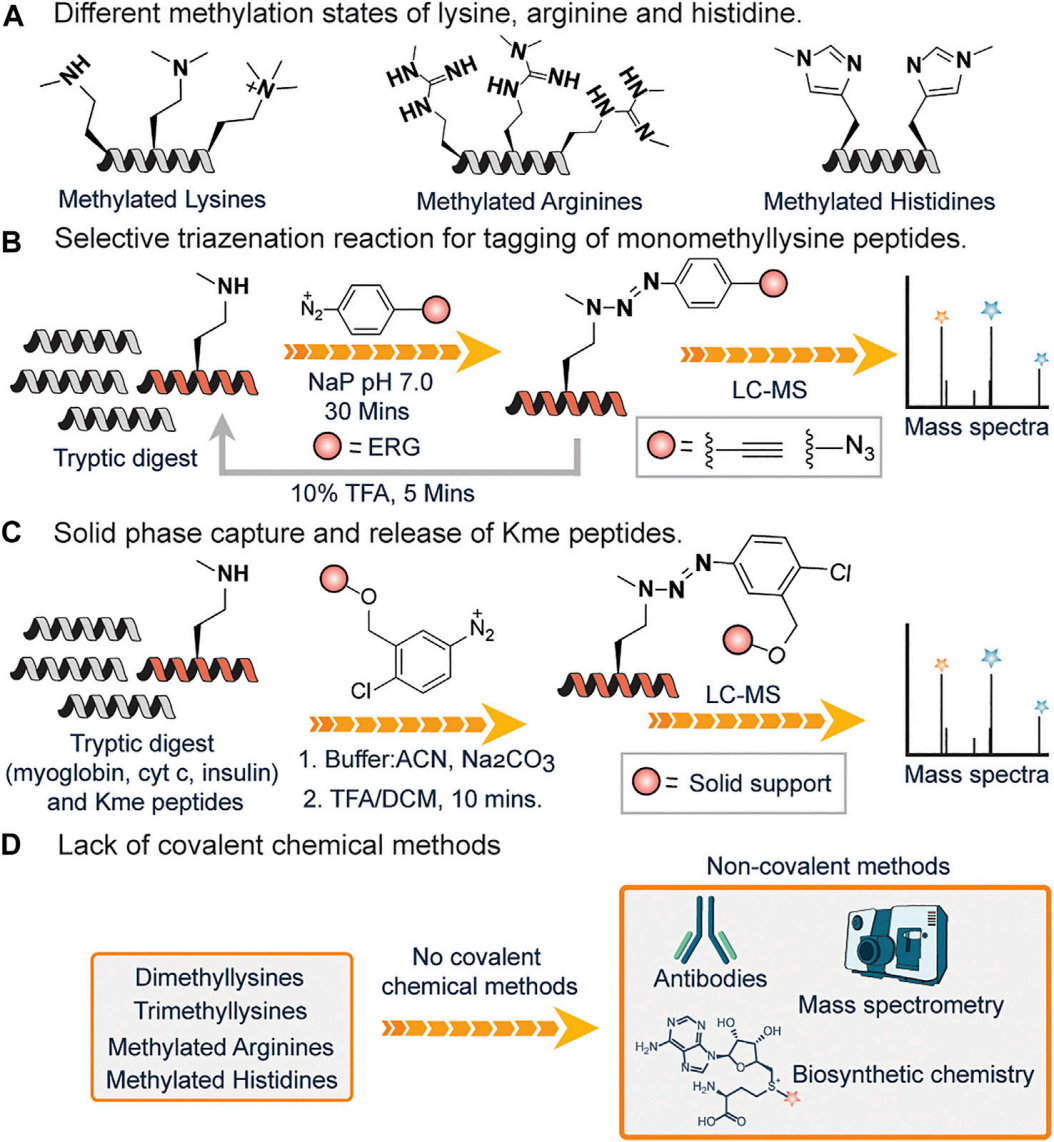
**(A)** The different methylation states of lysine, arginine, and histidine. **(B)** A selective triazenation reaction for labeling and enriching monomethyl lysine KMe post-translational modifications. **(C)** On-resin capture of KMe peptides from a tryptic digest of myoglobin, cytochrome c, and insulin spiked with KMe peptides. **(D)** Lack of covalent chemical strategy for tagging of dimethyl lysine, trimethyl lysine, methylated arginines and histidines.

Protein methylation typically occurs on lysine or arginine and, to a lesser extent, on histidine amino acid residues in proteins (Figure 6A). Lysine can be methylated one, two or three times by lysine methyltransferases, with different degrees of methylation leading to different functions and locations within a cell (Han et al., 2019). Arginine can be methylated once (monomethylated arginine) or twice, either with both methyl groups on one terminal nitrogen (asymmetric dimethylarginine) or one on both nitrogen’s (symmetric dimethylarginine), by enzyme arginine methyltransferases (PRMTs) (Yang and Bedford, 2013). Histidine can be methylated at two different positions either 1st or 3^rd^ position and recently has been shown to play important role in biological processes (Jakobsson, 2021) (Figure 6A).

Compared to other PTMs, the development of chemical tools for the detection of methylated residues is quite complicated, since the addition of a methyl group does not lead to a significant change in the physicochemical properties (charge and size) of proteins or peptides. So far there is only one chemical method for the pan-selective covalent modification of monomethyllysines (KMe) developed by our group. In 2020, our laboratory developed a triazenation reaction, which enables chemoselective labeling of KMe and enrichment from a complex mixture followed by subsequent chemically triggered decoupling to generate the native, unchanged coupling partners under mild conditions (Figure 6B) (Nwajiobi et al., 2021). By using electron-donating groups (EDG) on arene diazonium salts, selective modification of KMe peptides to triazenation products was observed within 30 min at pH 7.0 under mild reaction conditions. Although arene diazonium salts have been reported to react with primary amines, the reaction is reversible thus does not allow the enrichment and analysis of KMe peptides (Sonousi and Crich, 2015; Sengupta and Chandrasekaran, 2019). Similarly, the reaction of arene diazonium salts with tyrosine generated azo-coupling product, but it requires electron-withdrawing group (EWG) on arene diazonium ring and a slightly basic pH (∼9.0) conditions for efficient labeling (Schlick et al., 2005). Under our reaction conditions at pH 7 with ERG on the arene diazonium ions, we observed the chemoselective, pan-specific and near-quantitative modification of KMe peptides only within 30 min. In an attempt to achieve a traceless release of triazene products, we observed that the incubation of modified peptides in 10% trifluoroacetic acid (TFA) in water led to the decoupling of triazene products to unmodified KMe peptides in a traceless manner. This observation led to the application of this strategy for solid-phase capture of monomethyl lysine KMe peptides and the release of highly pure peptide samples for proteomics (Figure 6C). To this effect, arene diazonium ion functionalized resin was used to selectively capture and release KMe peptides from a complex tryptic digest mixture of myoglobin, cytochrome c (cyt c), and insulin.

In contrast to monomethyl lysine KMe, there are no covalent chemical methods for selective labeling of dimethyl and trimethyl lysines (Figure 6D). Existing non-covalent methods include the use of antibodies, mass spectrometry, stable isotope labeling techniques such as SILAC, and biosynthetic methods. Similarly, there are no covalent chemical methods for labeling methylated arginine and histidine residues.

### 3.5 Citrullination

Citrullination, also known as deimination, is an irreversible reaction that converts the guanidinium group of arginine to an ureido group, which results in the loss of both a positive charge and two potential hydrogen bond donors (Slade et al., 2014). The citrullination reaction is catalyzed by the protein arginine deiminases (PADs) and inhibits the methylation of the same arginine residue (Slade et al., 2014). The PADs have been documented to play a role in eukaryotic gene expression and are implicated in human diseases such as rheumatoid arthritis (Schellekens et al., 1998), multiple sclerosis (Musse et al., 2008), cancer (Chang et al., 2009) and inflammatory diseases (Kinloch et al., 2008). The detection, isolation, and enrichment of citrullinated proteins are challenging using MS and MS/MS analysis because the small 1 Da increase in mass imparted to the citrullinated protein is negligible. However, the following chemical tools have been developed for studying protein citrullination.

**FIGURE 7:**
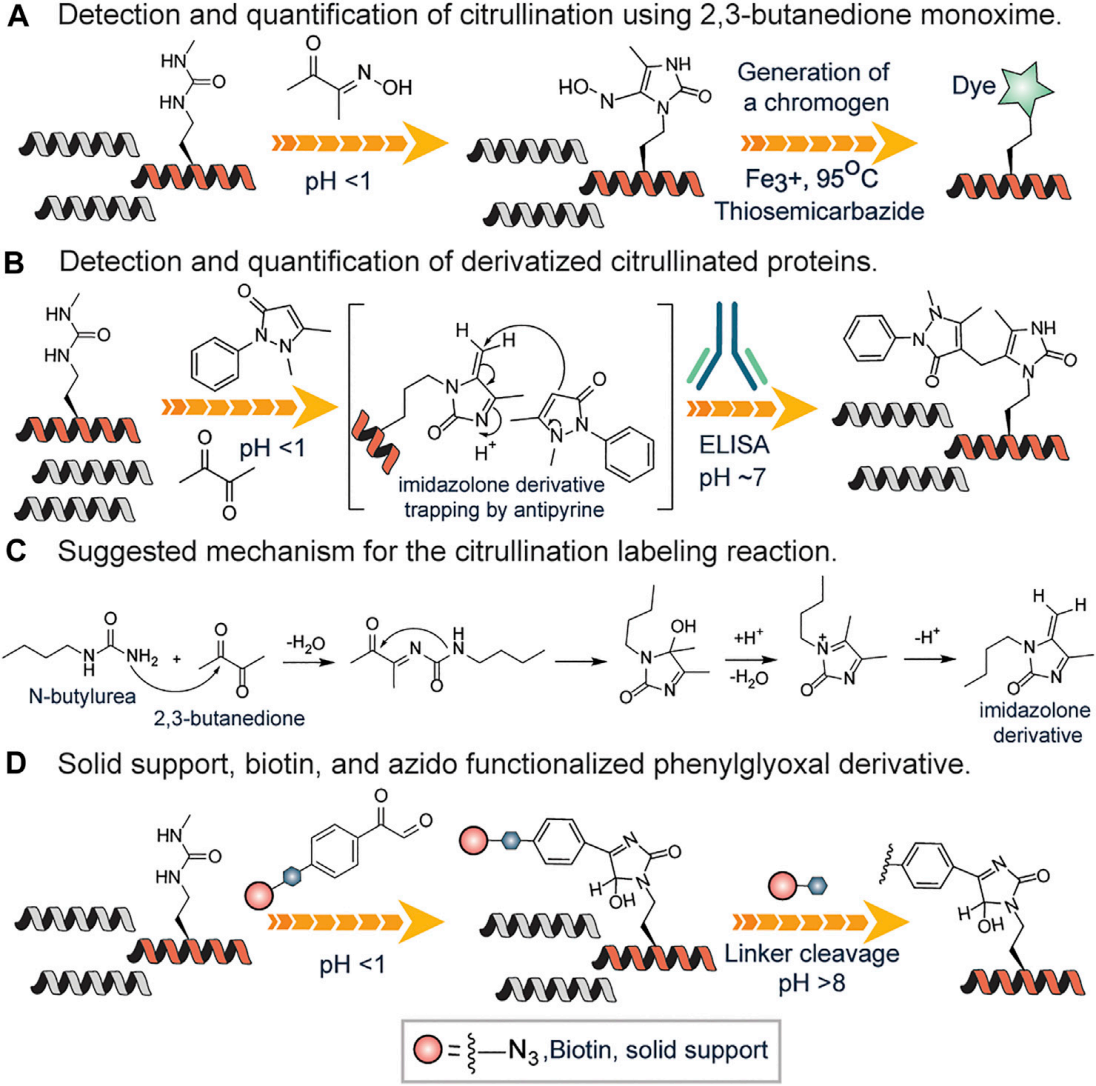
**(A)** Color development reagent assay of citrullination using 2,3-butanedione monoxime. **(B)** Antibody-based approach for detection and quantification of citrullination using antipyrine and 2,3-butanedione. **(C)** Proposed mechanism for the reaction of N-butyl urea with 2,3-butanedione, leading to the formation of the reactive imidazoline intermediate. **(D)** Solid-phase or affinity-based methods of detection and quantification of citrullinated proteins.

The color development reagent (COLDER) assay is commonly used to monitor citrullination levels (Knipp and Vašák, 2000). Many research groups have used the same approach to study arginine mimetics, peptides, and even proteins (Chumanevich et al., 2011; Willis et al., 2011; Bicker et al., 2012; McElwee et al., 2012). In this cost-effective and fast assay, the ureido group reacts with 2,3-butanedione monoxime to form an imidazoline which is stabilized by thiosemicarbazide (TSC) and Fe^3+^ facilitating rapid color development at 95^O^C (Knipp and Vašák, 2000) (Figure 7A). This method has a relatively high limit of detection (0.6 nM) thus unable to detect low abundant citrullinated proteins in the proteome. However, it has been used for evaluating PAD invitro activity (Slade et al., 2014). Proost *et al.* developed a chemically modified antibody-based method to specifically detect and quantify citrullination on proteins (Moelants et al., 2011). First, using antipyrine and 2,3-butanedione at low pH, the citrullinated proteins were chemically modified and subsequently detected and quantified by specific antibodies raised against a modified citrulline-containing peptide (Figure 7B). Citrulline-containing chemokine, i.e., CXCL8, was used as a model protein. This assay was developed into a commercially available Anti-Citrulline (Modified) Detection Kit (ACM kit; Millipore; Billerica, MA).

Fleckenstein and coworkers described the details of the chemistry behind the reaction of the ureido group of citrulline with 2,3-butanedione in the presence of antipyrine N-butyl urea as a model substance (Holm et al., 2006). They proposed that N-butyl urea reacts with 2,3-butanedione affording the reactive imidazoline derivative, which is further trapped by antipyrine as a nucleophile (Figures 7B,C). Also, they contended that acidic conditions are required not only to catalyze the nucleophilic addition of antipyrine but also to prevent arginine residues from reacting. Tutturen *et al.* demonstrated that phenylglyoxal modified citrulline under acidic conditions. They made a chemically cleavable phenylglyoxal derivative on PL-DMA resin and utilized it to enrich citrullinated proteins (Tutturen et al., 2010) from a complex mixture (Figure 7D). However, this approach is not chromophoric thus cannot be employed to visualize proteins. To surmount this challenge, Thompson and colleagues synthesized phenylglyoxal compounds with an azido group (Rh-PG) on the meta position for further reaction with alkyne modified rhodamine via click chemistry (Bicker et al., 2012) (Figure 7D). The Rh-PG labeling method was found to be better than the ACM kit: takes significantly less time (∼3 h versus ≥25 h), requires fewer steps (6 versus 12), and has simpler analysis (fluorescent imaging versus Western blotting). These studies showed that the Rh-PG probe could be a powerful chemical probe for detecting protein citrullination and for providing powerful insights into the role of specific PAD substrates in PAD-related diseases.

### 3.6 Lipidation

Lipidation is a common post-translational modification in which lipid moieties are covalently attached to proteins. It has been reported that at least six types of lipids, including fatty acids, isoprenoids, sterols, phospholipids, glycosylphosphatidylinositol (GPI) anchors and lipid-derived electrophiles (LDEs) can covalently modify proteins (Chen et al., 2018). These saturated and unsaturated fatty acids can attach to the lysine, serine or more commonly cysteine residues of proteins, in a process known as fatty acylation (Chen et al., 2018). Fatty acylation significantly increases the hydrophobicity of proteins leading to changes in their conformation, stability, membrane association, localization, trafficking, and binding affinity to their co-factors (Chen et al., 2018). Thus, the lipidation of proteins regulates protein-membrane interactions, protein-protein interactions, enzymatic activities, and protein stability (Jiang et al., 2018). However, aberrant protein lipidation is implicated in various diseases, including cancers, neurological disorders, and metabolic disorders (Chen et al., 2018). Hence, chemicals methods to detect lipidation PTM are essential to fully understand their roles in varying pathologies.

**FIGURE 8:**
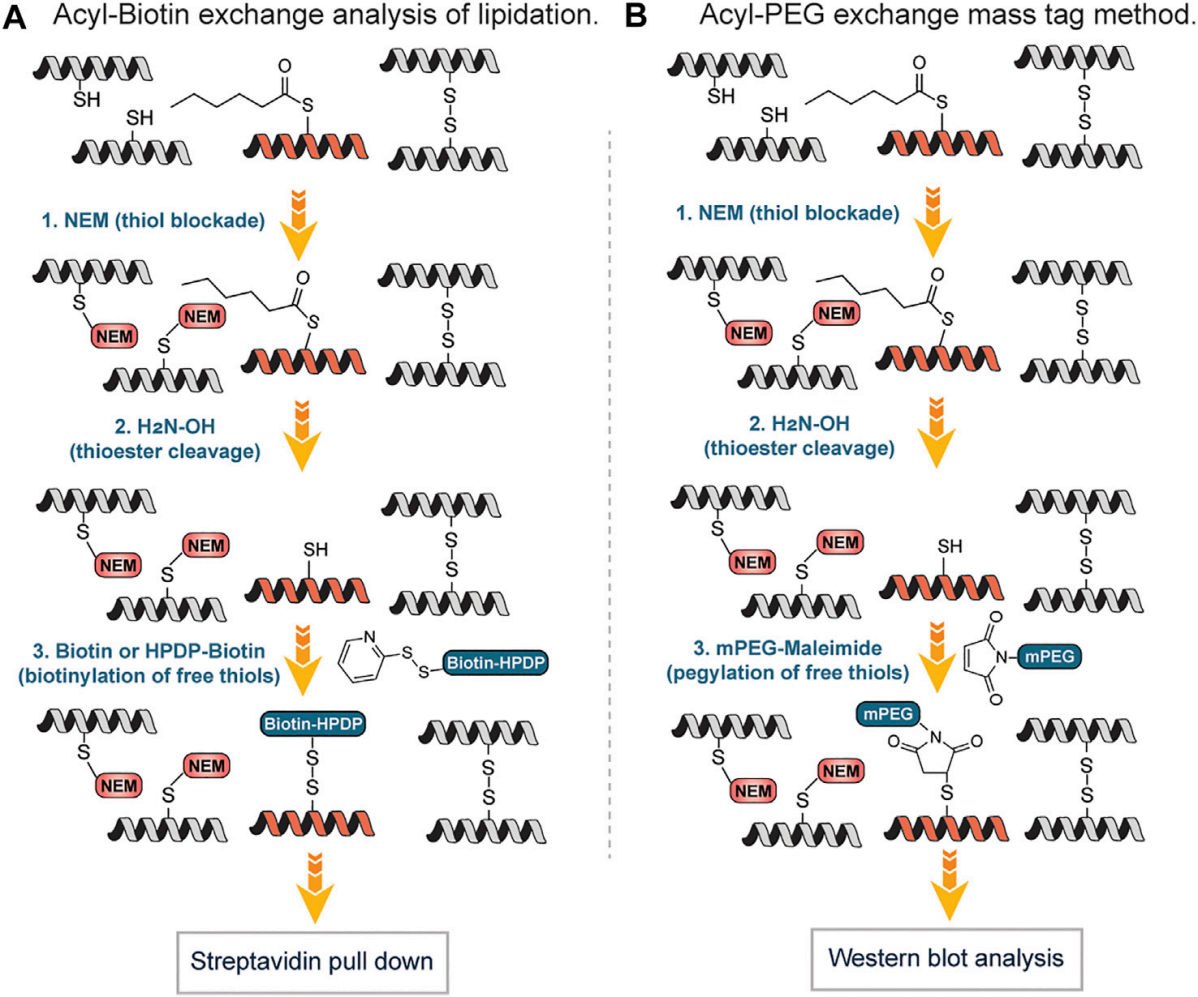
**(A)** Workflow for S-lipidation detection and quantification using acyl-biotin exchange (ABE) mediated approach. **(B)** Mass-tag labeling approach of S-fatty acylation using acyl-PEG exchange (APE).

Drisdel and Green described a method popularly known as Acyl-Biotin Exchange (ABE). Acyl-biotin exchange (ABE) remains one of the earliest chemicals methods developed to detect S-acylation of cysteines, based on the high reactivity of the thioester bond, which can be readily removed by weak bases, such as hydroxylamine (Drisdel and Green, 2004). It involves three sequential steps: blockade of free thiols with N-ethylmaleimide (NEM) followed by hydroxylamine-mediated cleavage of the palmitoyl-thioester bond to generate free thiols which are biotinylated with sulfhydryl-reactive reagents and enriched using streptavidin affinity purification (Figure 8A). Wan *et al.*, described a variation of this approach by using N-[6-(Biotinamido) hexyl]-3`-(2′-pyridyldithio) propionamide (Biotin-HPDP), instead of the 1-BiotinaMido-4-[4`-(MaleiMidoMethyl) cyclohexanecarboxaMido] butane (Biotin-BMCC) originally used by Drisdel and Green involving coupling of thiol with a maleimide moiety. Unlike Biotin-BMCC, Biotin–HPDP forms disulfide bonds with the thiols exposed following hydroxylamine treatment and facilitates an easy uncoupling of biotinylated proteins bound to the streptavidin–agarose affinity matrix, through *β*-mercaptoethanol (BME)-mediated cleavage of the biotin-Cys linkage (Wan et al., 2007) (Figure 8A). Also, Biotin–HPDP is more thiol specific as compared to maleimide of Biotin-BMCC that can react with the amine of lysine side chains. The ABE provides a highly sensitive method for the visualization and quantitative estimate of protein S-acylation thus enabling the global analysis of protein S-palmitoylation in yeast (Lei et al., 2022). S-acylated species usually do not move differently from the non-acylated proteins on protein gel electrophoresis since S-acylation does not alter the protein mass significantly and the electric charge remains the same on both species. Thus, it is difficult to directly detect the levels and the stoichiometry of acylation of a protein of interest by gel electrophoresis (Chen et al., 2018). To overcome this challenge and efficiently measure the endogenous levels of S-fatty acylation, Percher *et al.* developed a mass-tag labeling method termed acyl-PEG exchange (APE) involving cleavage of thioesters by hydroxylamine to generate free thiols followed by reaction with maleimide-functionalized polyethylene glycol reagents (Percher et al., 2016). The mass-tag induces a shift on the S-acylated proteins thus target proteins can be monitored by Western blot without the need for metabolic labeling or affinity enrichment of proteins (Figure 8B). With the APE approach, the endogenous levels of interferon-induced transmembrane protein 3 (IFITM3) S-fatty acylation were characterized. Equally, Forrester et al., found an alternative to the use of biotin and repeated protein precipitations in ABE, which detects S-acetylated proteins by using resin-assisted captured termed acyl-RAC (Forrester et al., 2011). In this approach, the free thiols were first blocked with MMTS. Secondly, thioesters were cleaved with neutral hydroxylamine (NH_2_OH) to generate thiols followed by capturing on thiol-reactive Sepharose resin. Employing “on-resin” proteolysis on the captured proteins, individual sites of S-acylation were identified. The method is fast and amenable to mass spectrometry techniques for identifying S-acylation sites.

One of the limitations of both ABE and APE approaches is the inability to identify the original acyl groups attached to the proteins since they are lost during the exchange step. Several research groups have surmounted this challenge using reporter groups that can be taken up by the cell, converted to acyl-CoAs and serve as lipid donors in cells. Hang *et al.*, employed ω-azido-fatty acids as probes for the rapid detection of protein fatty acylation in mammalian cells (Hang et al., 2007). After the metabolic labeling of mammalian proteins with the reporter groups, a bio-orthogonal reaction with a phosphine-biotin reagent via the Staudinger ligation was carried out to detect fatty-acylated protein. The labeled proteins were rapidly visualized with streptavidin-biotin affinity binding. Using the approach, monounsaturated fatty acylation, prenylation and N- or O-acylation, which could not be detected by acyl exchange methods were detected. Also, Charron et al. widened the applicability of this approach by using fluorescent chemical reporters that afforded a rapid biochemical analysis as well as imaging of protein fatty-acylation in mammalian cells (Charron et al., 2009).

### 3.7 S-Nitrosylation

S-nitrosylation is a posttranslational modification involving a covalent bond between a cysteine thiol and an NO equivalent. S-nitrosylation has been reported to play important functions in different biological processes including blood flow (Singel and Stamler, 2005), phosphorylation (Hess and Stamler, 2012), neuronal function (Lipton et al., 1993), transcriptional regulation (Marshall et al., 2000) and nitric oxide cellular signal transduction (Miersch and Mutus, 2005) while dysregulation of S-nitrosylation has been implication in cardiovascular diseases (Chung and David, 2010), cancers (Wang, 2012) and neurodegenerative diseases (Chung and David, 2010).

**FIGURE 9:**
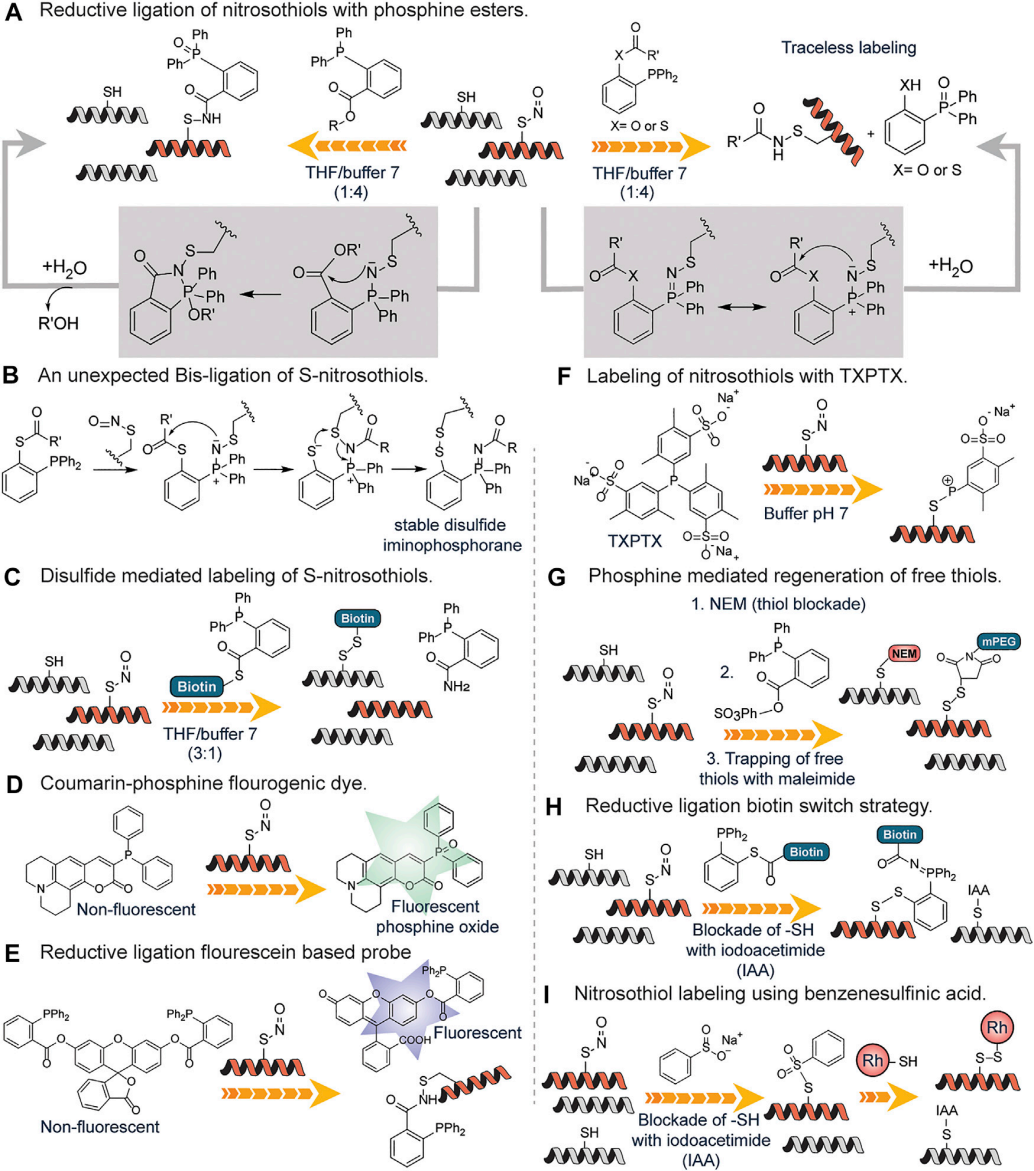
**(A)** Detection of S-nitrosylation through a fast reductive one-step ligation to generate sulfenamide conjugate with the phosphine oxide attached and the use of a traceless method which does not contain the phosphine oxide moiety. **(B)** Proposed reaction mechanism for the unexpected Bis-ligation reaction of S-nitrosothiols with phosphine esters. **(C)** Phosphine-thioester mediated conversion of unstable nitrosothiols to stable disulfides using a biotin functionalized probe. **(D)** Activation of the S-nitrosylation sensitive coumarin-phosphine probe upon oxidation by nitrosothiols found on proteins. **(E)** A more sensitive fluorescein-based probe for nitrosothiols with deacylation leading to turn-on of fluorescence. **(F)** Generation of stable S-alkylphosphonium ions through the reaction of nitrosothiol containing peptides with triarylphosphines (TXPTS) in water. **(G)** Phosphine reagent mediated over the reduction of sulfenamide to generate free thiols that are trapped using maleimide-based chemistry. **(H)** Formation of peptide-probe disulfide bond through the reductive ligation of a phosphine-biotin thioester probe onto nitrosothiols. **(I)** S-nitrosylation labeling using benzene sulfinic acid followed by the subsequent trapping with a rhodamine-thiol-based probe to generate a stable disulfide linkage.

The detection of S-nitrosylation poses a challenge due to the labile nature of S-nitrosothiols. Wang and Xian developed a fast reductive one-step ligation reaction that targets SNO moieties converting the unstable S-nitrosothiols into stable sulfenamide conjugates by phosphine esters (Wang and Xian, 2008) (Figure 9A). The reaction mechanism is believed to be similar to the Staudinger ligation of azides (Lin et al., 2005). The same research group developed a traceless version of this reaction in which an amide bond is formed between S-nitrosothiols and phosphine ester/thioester conjugates without the phosphine oxide moiety (Zhang et al., 2009a) (Figure 9A). This approach was inspired by the well-known traceless Staudinger ligation (Nilsson et al., 2000). They also reported an unexpected bis-ligation reaction of RSNOs triaryl substituted phosphine-thioester substrates that converts unstable primary RSNOs to stable disulfide-iminophosphorane products in good yields under mild conditions (Zhang et al., 2009b) (Figure 9B). In contrast to the sulfenamide products from the reductive ligation of RSNOs, disulfide-iminophosphorane products were not sensitive to phosphine reagents.

They went further to develop a reductive ligation-mediated disulfide formation between RSNOs and phosphine-thioester substrates which selectively converts unstable RSNOs to stable disulfides (Zhang et al., 2010) (Figure 9C). This approach was used in identifying SNO proteins in cell extracts. To increase the utility of the reductive ligation to detect S-nitrosylation, Pan et al. developed the first generation of fluorescent probes for SNO, which was based on SNO-mediated oxidation of phosphine substrates in which a coumarin-phosphine compound was used to measure the concentration of S-nitrosothiols (Pan et al., 2009) (Figure 9D). The coumarin-phosphine serves as a fluorogenic dye that is activated by S-nitrosothiols. This assay showed greater sensitivity to SNO but other reactive sulfur species could undergo oxidation as well (Zhang et al., 2014). To circumvent this, Zhang et al. developed a more sensitive SNO specific fluorogenic probe based on reductive ligation of SNO called SNOP1 (Zhang et al., 2014) (Figure 9E). The probes were based on the well-known principle that acylation on many fluorophores can quench the fluorescence and de-acylation can reform the fluorescent species (Li et al., 2014). Thus, hydroxyl (-OH) sensitive fluorophore was acylated to the triaryl phosphine probe and upon reacting with SNO, the fluorophore is released serving as a more sensitive means of probing for SNO. This fluorogenic probe works via a similar pathway as mentioned in Figure 9A. The applicability of this approach was demonstrated for the detection of GSNO in diluted deproteinized bovine plasma thus excellent for sensitive analysis of GSNO in biological samples (Li et al., 2014).

King and coworkers detailed the first reaction of triarylphosphines with RSNOs in water (Bechtold et al., 2010) (Figure 9F). The water-soluble triarylphosphine TXPTS reacted directly with S-nitrosothiol residues and generated stable S-alkylphosphonium ions, TXPTS-derived aza-ylide as well as TXPTS oxide. The formation of stable S-alkylphosphonium ions is due to the presence of sulfonate groups on the aromatic ring of triaryl phosphine. They showed efficient labeling of S-nitrothiols, S-nitrosoglutathione and a mutated peroxiredoxin, S-nitrosated C165S alkyl hydroperoxide reductase (C165S AhpC-SNO). This represented the first covalent detection and labeling of S-nitrothiols on proteins in the buffer.

Shao *et al.*, developed a fluorogenic probe, termed PSNO, for the *in-situ* imaging of protein S-nitrosylation in live cells based on phosphine-mediated reductive ligation. In this reaction, endogenous thiols were first blocked followed by conversion of SNO to free thiol by phosphine-mediated reductive ligation and labeling with maleimide PEG. S-nitrosoglutathione (GSNO) was employed as a model substrate and an array of phosphine-based reagents were tested for their reactivity towards it. They found that PSNO was highly sensitive and specific towards GSNO in buffer, and they successfully imaged protein S-nitrosylated GAPDH in live cells (Shao et al., 2017).

Li *et al.* took advantage of the finding that the sulfenamide intermediate produced from reductive ligation can be over-reduced in the presence of excess phosphine reagent resulting in the formation of free thiol from the starting S-nitrosothiols. They developed a strategy to target these free thiols (Li et al., 2012) (Figure 9G). To forestall cross-reactivity with endogenous thiols, N-ethyl maleimide (NEM) was used to block all free thiols. Following thiol blockade, triphenylphosphine ester derivatives were used to selectively reduce SNO bonds in proteins. They demonstrated that triphenylphosphine ester derivatives are specific reductants of SNO in complex biological samples without the reduction of protein disulfides or protein thiols modified by hydrogen peroxide. The thiols generated from the reduction step were tagged with biotin or fluorescently labeled maleimide reagents. Using this approach, they efficiently labeled protein S-nitrosylation in both extracts and whole fixed cells without reducing protein disulfides.

Seneviratne et al. strategically combined reductive ligation reaction with biotin switch strategy in a reaction they coined SNOTRAP (SNO trapping by triaryl phosphine) which enhanced identification of S-nitrosylated proteins by mass spectrometry (Seneviratne et al., 2016). The SNOTRAP probe is made up of a triphenylphosphine thioester linked to a biotin molecule with polyethylene glycol (PEG) spacer group serving as a linker (Figure 9H) The triphenylphosphine thioester probe reacted with SNO groups and afforded an azaylide intermediate, which through a well-positioned electrophile (thioester), rearranged to form a disulfide–iminophosphorane similar to Figure 9B. The assay is in three steps: blocking of free cysteine residues with iodoacetamide (IAA) followed by biotin-mediated affinity capture of disulfide–iminophosphorane and release of S-nitrosylated proteins and nanoflow liquid chromatography-MSMS (nLC-MS/MS) analysis (Figure 9H). They applied this approach in fully assessing protein S-nitrosylation in the neurodegenerating brain where they discovered that S-nitrosylation is increased during the early stages of neurodegeneration.

Reeves *et al.* examined in greater detail the conversion of S-nitrosothiols to thiosulfonates with the sodium salt of benzenesulfinic acid which was first reported by Hart in 1985 (Hart, 1985; Reeves et al., 2013; Reeves et al., 2014) (Figure 9I). While Hart carried out the reaction under very acidic conditions, the results showed that the reaction is feasible under modestly acidic conditions (pH 4.0) making it biocompatible. S-nitrosoglutathione (GSNO) and S-nitrosylated bovine serum albumin (SNO-BSA) were selectively trapped by benzenesulfinic acid sodium salt under acidic conditions (Figure 9I). Though PhSO_2_Na was successfully employed as an S-nitrosothiol probe, the S-phenyl sulfonyl cysteine functional group, the resultant linkage formed at the point of conjugation underwent hydrolysis at neutral pH resulting in the diminished signal. To salvage this, Reeves *et al.* developed a protocol coined thiosulfonate switch that displaced S-phenylthiosulfonates with a thiol-containing fluorescent probe to generate a stable disulfide linkage. The protocol as described had three steps: blockage of free cysteines using S-phenylsulfonylcysteine (a thiol blocking reagent, that blocks free thiols at pH 4.0 in the presence of S-nitrosothiols); addition of PhSO_2_Na to convert protein SNO into protein S-phenylthiosulfonates and subsequent introduction of rhodamine based fluorophore possessing a reactive thiol to facilitate a displacement reaction with the protein S-phenylthiosulfonates to afford a disulfide between the probe and the formerly S-nitrosylated cysteine residue (Figure 9I). The purified proteins BSA-SNO, and alcohol dehydrogenase regulator (AdhR*-SNO) were labeled using this technique without the generation of false positive characteristics of reductive techniques since it is not dependent on the reduction of S-nitrosothiol to a free thiol. Other methods of labeling S-nitrosylation including the famous biotin switch technique and other cysteine oxidation products are well covered in these reviews (Chung et al., 2013; Alcock et al., 2018).

## 4 Perspectives and Concluding Remarks

In summary, tremendous progress has been made in the past several years in developing PTM-specific enrichment methods, and MS-based proteomics technologies for PTM analysis but chemical approaches for the selective labeling of PTM for enrichment are still in their infancy. Given the high number of PTMs (for example, phosphorylation, ubiquitination, and lysine acetylation, methylations), PTMs likely constitute the most complex and delicate regulatory networks and are involved in controlling various biological functions. Any changes/abrasion in these PTMs lead to a variety of different diseases thus detecting each PTM and its crosstalk with other PTMs is necessary to understand the biological processes and for the development of new therapies. Current attempts at elucidating the crosstalk between PTMs have relied heavily on PTM proteomics. Villen *et al.* recently described in detail the advances in proteome-wide identification of PTM crosstalk through the use of proteomics technologies, mass spectrometry instrumentation, and bioinformatics (Leutert et al., 2021).

The challenges with PTM proteomics using MS and enrichment-based methods include lower sensitivity for the detection of low-abundance PTMs, poor accuracy in PTM identification and localization, and lack of methods for the analysis of all types of PTMs in proteins (Hermann et al., 2022). One of the ways to tackle the challenges associated with PTM proteomics is by developing highly chemoselective pan-specific chemical probes that are capable of forming strong covalent bonds with a modified protein independent of the amino acid sequence. These chemical methods would label post-translational modified proteins (including low abundance) with affinity groups that can be enriched and analyzed by MS. Such chemical strategies will allow detecting modifications on proteolytic peptide fragments and also in regulatory protein domains and intact proteins. Pan-specific chemical methods for each type of PTMs would enable the identification of full-spectrum PTMs and multiple PTMs in proteins to understand the cooperative PTM events in proteins.
